# IL-21 Rescues the Defect of IL-10-Producing Regulatory B Cells and Improves Allergic Asthma in DOCK8 Deficient Mice

**DOI:** 10.3389/fimmu.2021.695596

**Published:** 2021-11-15

**Authors:** Jinqiu Jiang, Tao Qin, Liang Zhang, Qiao Liu, Jiabin Wu, Rongxin Dai, Lina Zhou, Qin Zhao, Xiaoyan Luo, Hua Wang, Xiaodong Zhao

**Affiliations:** ^1^ Department of Dermatology, Ministry of Education Key Laboratory of Child Development and Disorders, National Clinical Research Center for Child Health and Disorders, China International Science and Technology Cooperation Base of Child Development and Critical Disorders, Children’s Hospital of Chongqing Medical University, Chongqing, China; ^2^ Chongqing Key Laboratory of Child Infection and Immunity, Children’s Hospital of Chongqing Medical University, Chongqing, China; ^3^ Department of Infectious Diseases, Children’s Hospital of Chongqing Medical University, Chongqing, China; ^4^ Department of Rheumatology and Immunology, Children’s Hospital of Chongqing Medical University, Chongqing, China

**Keywords:** regulatory B cells, IL-10, DOCK8 deficiency, asthma, IL-21

## Abstract

Mutations in human DOCK8 cause a combined immunodeficiency syndrome characterized by allergic diseases such as asthma and food allergy. However, the underlying mechanism is unclear. Regulatory B (Breg) cells that produce IL-10 exert potent immunosuppressive functions in patients with allergic and autoimmune disorders. DOCK8-deficient B cells show diminished responses to TLR9 signaling, suggesting a possible defect in IL-10-producing Breg cells in those with DOCK8 deficiency, which may contribute to allergies. Here, we isolated peripheral blood mononuclear cells from DOCK8-deficient patients and generated a Dock8 KO mouse model to study the effect of DOCK8 deficiency on Breg cells. DOCK8-deficient patients and Dock8 KO mice harbored quantitative and qualitative defects in IL-10-producing Breg cells; these defects were caused by abnormal Dock8^-/-^ CD4^+^ T cells. We found that recombinant murine (rm)IL-21 restored the function of Bregs both *in vitro* and in Dock8 KO mice, leading to reduced inflammatory cell infiltration of the lungs in a murine asthma model. Overall, the results provide new insight into the potential design of Breg-based or IL-21-based therapeutic strategies for allergic diseases, including asthma associated with DOCK8 deficiency.

## Intoduction

Mutations in dedicator of cytokinesis 8 (DOCK8) are the major cause of autosomal recessive hyper-IgE syndromes (HIES), which are characterized by combined immunodeficiency and elevated serum IgE levels (1). Patients with HIES caused by DOCK8 deficiency are more susceptible to developing allergic diseases (e.g., asthma, food allergies, and atopic dermatitis) than those with HIES caused by STAT3 mutations (2). Recent work identified some of the mechanisms underlying the high incidence of allergy in DOCK8 deficiency. For example, Dock8^-/-^ IL-13^hi^ IL-4^hi^ IL-5^hi^ IL-21^lo^ follicular helper T (Tfh)13 cells are associated with production of high-affinity IgE antibodies (3), and migration-induced cell shattering causes a type 2-biased helper T cell response (4). However, the role of DOCK8-deficient B cells in the pathogenesis of allergic diseases remains unclear.

DOCK8 functions as a guanine nucleotide exchange factor that is important for actin cytoskeleton rearrangement and optimal STAT3 phosphorylation; it also serves as an adaptor molecule for TLR9-MYD88 signaling in B cells (5, 6). Loss-of-function mutations in DOCK8 contribute to impairment of B cell function and of long-lived memory responses (7).

B cells play an important role in the pathogenesis of allergic diseases, in particular by secreting IgE. However, regulatory B (Breg) cells in humans and mice are defined as B cells with immunosuppressive capacity associated with secretion of anti-inflammatory cytokines such as TGF-β, IL-35, and, particularly, IL-10 (8–11). Several Breg subsets exert immunoregulatory functions in allergic and inflammatory diseases by secreting IL-10; these cells, such as mouse CD1d^hi^CD5^+^ B cells (termed B10 cells) (12), CD21^+^CD23^-^ marginal zone (MZ) B cells (13), and human CD19^+^CD24^hi^CD27^+^ memory B cells (14), and CD19^+^CD24^hi^CD38^hi^ immature transitional B cells (15) share partially overlapping phenotypes. IL-10-producing Breg cells have direct and indirect suppressive effects on proliferation and cytokine production by effector T cells. With respect to the signaling mechanisms underlying development of IL-10-producing Breg cells, previous studies report that BCR-derived signals initiate acquisition of regulatory B10-like competence. Then, LPS-induced TLR4 and TLR9 signaling facilitates a transcriptionally active conformation of the *IL10* gene in pro-B10 cells (16, 17); also, activation of STAT3 is required for TLR-induced IL-10 production by B cells (18, 19). In addition, IL-21- and CD40-dependent cognate interactions with T cells are required to generate fully functional mouse B10 cells (20). DOCK8-deficient B cells show diminished responses to TLR9 signaling, suggesting a possible defect in IL-10-producing Breg cells in those with DOCK8 deficiency, which may contribute to allergies.

IL-21 is a type I cytokine produced mainly by activated CD4^+^ T cells and natural killer T (NKT) cells (21, 22). Upon binding to its receptors (IL-21R and a common receptor γ chain), IL-21 activates the Janus family tyrosine kinases members JAK1 and JAK3, with subsequent phosphorylation of STAT3 and STAT1 (23). For functionality, IL-21 mediates maturation of B cells, normal development of T follicular helper cells, and differentiation of Th17 cells (24).

In this study, we generated Dock8 KO mice using the TALEN technique; these mice harbor a frameshift mutation in the first exon of *Dock8*, which mirrors human disease because most of the DOCK8-deficiency in patients is caused by the frame shift or gene deletion in different exons of the DOCK8 gene rather than by a point mutation. We found that DOCK8-deficient patients and Dock8 KO mice harbor both quantitative and qualitative defects in IL-10-producing Breg cells due to abnormalities in Dock8^-/-^ CD4^+^ T cell populations. We also found that IL-21 rescued the function of Bregs in Dock8 KO mice and alleviated inflammatory infiltration in a murine asthma model.

## Materials and Method

### Patients

Three Chinese patients with mutations in the DOCK8 gene were enrolled in the study. All patients were admitted to the Children’s Hospital of Chongqing Medical University. Diagnosis of the patients was described previously (25). Three age-matched subjects were enrolled as healthy controls (HCs). Informed consent to participate in the study was provided by the patients’ families, and the study was approved by the Medical Ethics Committee of Children’s Hospital of Chongqing Medical University.

### Mice

Dock8 KO mice were generated using the TALEN technique (Shanghai Biomodel Organism Science & Technology Development Co., Ltd). The first exon of *Dock8* was chosen for TALEN-induced mutagenesis; absence of a 45 bp sequence from the coding frame of exon 1 introduced a reading frame shift into the Dock8 gene (26). Dock8 was genotyped by PCR using the following primer pair: sense, 5’- GGGGGATCCCCTGCGGCCGGCGACTCTGA-3’, and antisense, 5’- GGGGAATTCGAAGCGGGGAAGGCAATGATGACA-3’. PCR products amplified from F0 generation mouse tail tissue were purified, cloned, and sequenced to identify positive founder mice with the Dock8 protein harboring the frame shift. Positive F0 generation mice were crossed with C57BL/6J mice and the genotype of the offspring was confirmed by PCR, cloning, and sequencing. CD4 KO and CD45.1^+^ C57BL/6 mice were obtained from the Jackson Laboratory. C57BL/6 mice were purchased from the Laboratory Animal Center, Chongqing Medical University. All mice were aged 6 to 10 weeks at the time of the experiments and were housed in specific pathogen-free animal facilities. Data were obtained from three or more mice per group. All animal experiments were reviewed and approved by the Institutional Animal Care and Usage Committee of Children’s Hospital of Chongqing Medical University.

### Flow Cytometry and Phosphorylation Analyses

Heparinized blood was obtained from patients and from age-matched HC subjects. Peripheral blood mononuclear cells (PBMCs) were isolated by Ficoll density gradient centrifugation and cell numbers were counted in a hemocytometer. Flow cytometry was performed using a FACSCanto II cytometer (BD Biosciences, San Jose, Calif). Briefly, PBMCs were stained with anti-human CD19-APC, anti-human CD24-PE, anti-human CD27-BV450, anti-human CD38-PerCP-cy5.5, or anti-human IgD-BV510. Mononuclear cells isolated from the spleen of DOCK8 KO and WT mice were stained with the following antibodies: anti-CD19 FITC, anti-CD5 PE, and anti-CD1d APC (CD1d^hi^CD5^+^ B cells); anti-CD19 FITC, anti-CD23 PE, anti-CD21 APC, and anti-CD24 Percp-cy5.5 (T2-MZP cells) (all antibodies were from BioLegend, CA). Mononuclear cells isolated from spleen cells of chimera mice were stained with anti-CD45.1 BV510, CD45.2 FITC, anti-CD19 APC, and anti-IL-10 PE (BioLegend, CA). Mononuclear cells isolated from the spleen of CD4 KO mice were stained with anti-CD19 FITC or APC, anti-CD5 PE, anti-CD1d APC, anti-CD23 PE, anti-CD21 APC, anti-CD24 Percp-cy5.5, or anti-IL-10 PE (BioLegend, CA). To detect phosphorylation, single-cell suspensions of splenocytes were stimulated for 3 h with lipopolysaccharide (LPS; 10 μg/mL, Sigma, St. Louis) or for 30 min with rmIL-21 (100 ng/mL; R&D Systems). Cells were then fixed and permeabilized with BD Phosflow Lyse/Fix Buffer and Perm Buffer III (BD Biosciences), respectively. Finally, cells were stained with anti-B220 FITC, anti-CD5 BV421, anti-CD1d APC, or anti-pY727 PE. All data were analyzed using FlowJo software.

### B Cell Stimulation

PBMCs isolated from patients or HCs were resuspended (at 2×10^6^ cells/mL) in 48-well flat-bottom plates in culture medium and stimulated for 7 h with LPS (10 μg/mL; Sigma, St. Louis), phorbol 12-myristate 13-acetate (PMA, 20 ng/mL; Sigma, St. Louis), ionomycin (1 μg/mL; Sigma, St. Louis), and brefeldin A (BFA, 1×solution/mL; BioLegend, CA) before staining and flow cytometry analysis. Next, the cells were harvested and washed twice with PBS. Single-cell suspensions were then stained for 20 min on ice with predetermined optimal concentrations of anti-CD19 APC (BioLegend, CA). Stained cells were washed twice with PBS before fixation and permeabilization in Fixation and Permeabilization Buffer (BioLegend, CA). Finally, cells were stained for 30 min with anti-IL-10 PE (BioLegend, CA). To detect B10 cells in mice, single-cell suspensions of splenocytes were stimulated for 5 h with LPS, PMA (50 ng/mL), ionomycin (500 ng/mL), and BFA, followed by staining with anti-CD19 APC (BioLegend, CA) and anti-IL-10 PE (BioLegend, CA). For co-culture experiments, splenic B cells were purified using an EasySep™ Mouse B Cell Isolation Kit (STEMCELL Technologies, Canada). The cells (2×10^6^ cells/mL) were then incubated for 48 h with mouse rmIL-21 (100 ng/mL, R&D Systems). After culture for 48 h, the IL-10 concentration in the supernatant was measured in an ELISA (BioLegend, CA), and B10 cell numbers were measured by flow cytometry analysis, as described above.

### Generation of Bone Marrow Chimeras

Bone marrow was collected from Dock8^-/-^ (CD45.2^+^) mice and from C57BL/6 wild-type (CD45.1^+^) mice. For each chimera, CD45.2^+^ wild-type or Dock8 KO bone marrow cells plus CD45.1^+^ bone marrow cells (4 × 10^6^ cells in a 1:1 mixture) were transferred intravenously into lethally-irradiated (two doses of 550 rads each) wild-type CD45.1^+^ recipients. Recipient mice were allowed 8 weeks to reconstitute prior to challenge with ovalbumin (OVA).

### Generation of OVA-Induced Allergic Asthma Model Mice and Nasal Administration of Recombinant IL-21

OVA-induced allergic asthma was elicited by sensitization with chicken OVA (5 μg, intraperitoneally (i.p.); Sigma-Aldrich) emulsified in 200 μl Imject Alum (Thermo Fisher Scientific) on Day 0, followed by two oropharyngeal aspiration challenges (on Days 14 and 21) with 1.5% OVA dissolved in 50 μl PBS. Control mice received only PBS. Mice were harvested 72 h after the second challenge (27). Some mice received 20 ng of rmIL-21 (R&D Systems) into the nostrils (daily on Days 15–17, 18–20, or 15–20). This 3 day protocol was decided by conducting preliminary time-course experiments. The dose of rmIL-21 was obtained from a previous study (28).

### Adoptive Cell Transfer

Naïve splenic CD4^+^ T cells isolated from Dock8 KO or wild-type mice were purified with the EasySep™ Mouse Naïve CD4^+^ T Cell Isolation Kit (STEMCELL Technologies, Canada). Next, 5×10^6^ naïve CD4^+^ T cells were adoptively transferred into CD4 KO mice *via* intravenous injection 1 day before immunization with OVA. OVA immunization was performed as described above. 100 μl blood was taken from the tail vein of CD4KO mice on day 7, day14 and day 21 to detect the number of CD4^+^ T cells. Recipient mice were analyzed on Day 24 post-OVA immunization.

### Histopathological Analysis

Lung tissue was harvested and fixed for 24 h in 10% formalin and then embedded in paraffin. Sections (4 μm) were stained with hematoxylin and eosin. The degree of airway infiltration by inflammatory cells was scored by two independent investigators by double-blind screening. Peri-bronchiole and peri-vascular inflammation were evaluated using a scoring system of 0–4, where 0 represents no cells; 1, a few cells; 2, a ring of inflammatory cells one cell layer deep; 3, a ring of inflammatory cells 2–4 cells deep; and 4, a ring of inflammatory cells >4 cells deep.

### Statistical Analysis

GraphPad Prism 5 software was used for statistical analyses. Results are expressed as the mean ± SEM. The significance of differences between groups was determined using a two tailed unpaired Student’s *t* test or ANOVA. A P value <0.05 was deemed statistically significant.

## Results

### Breg Subsets Are Present in DOCK8-Deficient Patients and Dock8 KO Mice, but Are Not Capable of Producing IL-10

**Figure 1:**
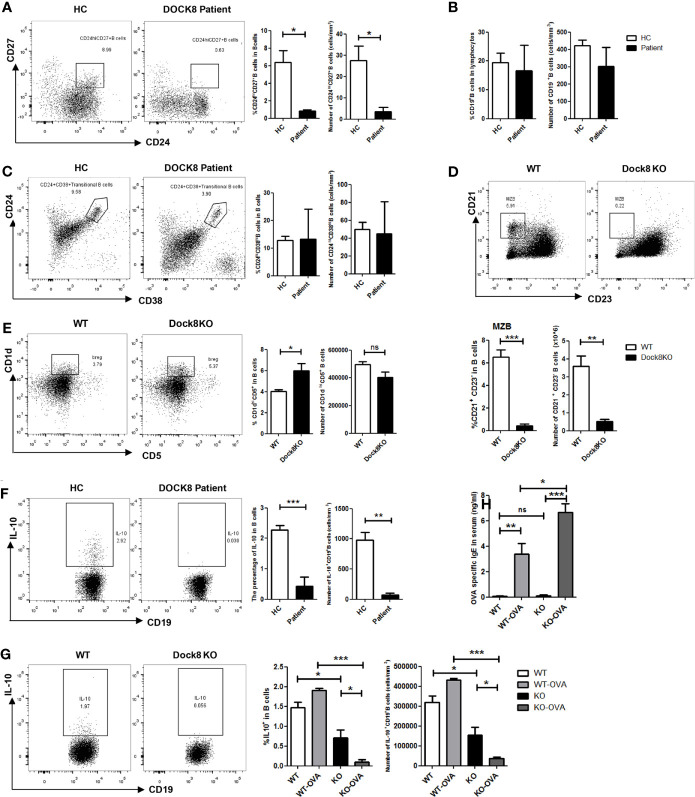
Reduced percentage of IL-10-producing Breg cells in DOCK8-deficient patients and Dock8 KO mice. **(A)** Percentage and number of CD19^+^CD24^hi^CD27^+^ B cells, **(B)** CD19^+^ B cells, and **(C)** CD19^+^CD24^hi^CD38^hi^ B cells in HCs and DOCK8-deficient patients (n = 3 for both). **(D)** CD21^+^CD23^-^ marginal zone B cells and **(E)** CD5^+^CD1d^hi^ B cell populations in the spleen of wild-type and Dock8 KO mice (n = 4 for both). **(F)** The percentage of IL-10^+^CD19^+^ Breg cells in HCs and DOCK8-deficient patients (n = 3 for both). **(G)** Percentage of IL-10^+^CD19^+^ Breg cells in the spleen of wild-type and Dock8 KO mice immunized (or not) with OVA (n = 4 for both). **(H)** OVA-specific IgE in serum of Dock8 KO mice immunized with OVA was measured by ELISA. *P < 0.05, **P < 0.01, and ***P < 0.001, and ns is not significant (Student’s *t* test). Data are representative of three independent experiments.

To investigate the effects of DOCK8 deficiency on homeostasis of Breg cells in humans, we isolated PBMCs from three patients (aged 5–14 years) with confirmed DOCK8 deficiency (**Table 1**). First, we examined Breg subsets. We found that the percentage and number of CD19^+^CD24^hi^CD27^+^ memory B cells within the PBMC population was lower in patients than in age-matched HCs (**Figure 1A**), whereas the total B cell (**Figure 1B**) and CD19^+^CD24^hi^CD38^hi^ immature transitional B cell populations (**Figure 1C**), which harbor IL-10-producing Breg cells, were normal. In Dock8 KO mice, analysis of splenic B cells revealed a near absence of CD21^+^CD23^-^ marginal zone B cells (**Figure 1D**) but a higher frequency of CD1d^hi^CD5^+^ B10 cells (**Figure 1E**) than in controls; these cell populations also harbor IL-10-producing Breg cells.

**Table 1:** Clinical features of DOCK8-deficient patients.

Features	P1/Male	P2/Female	P3/Male
Age at onset	6 y	1 y 7 m	3 y
Age at diagnosis	14 y	5 y 8 m	9 y
*DOCK8* mutation	Exon11 homozygous deletion + Exon 12–33 heterozygous deletion	Exon2 homozygous deletion + Exon1, 3–39 heterozygous deletion	c.1278-1279delTG, p.V427fsX435
DOCK8 protein expression	–	–	–
Atopy	Eczema, asthma	Eczema,	Eczema, food allergy
Immunodeficiency	Respiratory infection, otitis media, sinusitis, stomatitis	Respiratory infection, otitis media, stomatitis	Respiratory infection, otitis media, stomatitis
Autoimmune disease	–	–	Autoimmune hemolytic anemia
Malignancy	–	–	–
Etiology of infections	HSV	EBV	EBV
Molluscum contagiosum	+	+	+

HSV, herpes simplex virus; EBV, Epstein–Barr virus. ; −, negtive; +, positive.

Next, we examined whether DOCK8 expression affects the function of Breg cells in DOCK8-deficient patients and Dock8 KO mice. PBMCs from patients and HCs were stimulated for 7 h with LPS plus PMA, ionomycin, and BFA to determine whether the absence of DOCK8 affects IL-10 production by B cells. The results showed that IL-10^+^ B cell numbers in patients were significantly lower than those in HCs; indeed, they were almost absent from patients (**Figure 1F**). The percentage and number of IL-10^+^ B cells in Dock8 KO mice were also lower than those in wild-type mice, especially after immunization with OVA (**Figure 1G**). Previously, we reported that, compared with wild-type mice, Dock8 KO mice show increased serum IgE levels and develop significant airway inflammatory infiltration and airway hyper-responsiveness in the OVA-induced allergic asthma model ^20^, a finding that is consistent with the data presented above; the serum level of OVA-specific IgE in Dock8 KO mice was also increased (**Figure 1H**).

### DOCK8 Deficiency Causes a Partial Intrinsic Defect in Breg Cells

**Figure 2:**
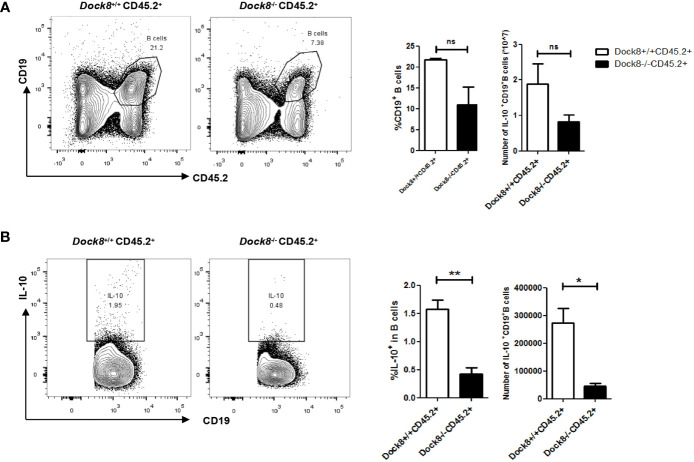
Breg defect in Dock8 deficiency is not entirely B cell intrinsic. **(A, B)** CD45.2^+^ wild-type or Dock8 KO bone marrow cells were transferred intravenously into lethally-irradiated CD45.1^+^ wild-type recipients (n = 3 for both) prior to immunization with OVA. Flow cytometry analysis of CD45.2^+^CD19^+^ B cells and CD45.2^+^CD19^+^IL-10^+^ B cells in the splenocyte population from CD45.1^+^ chimeric mice. *P < 0.05, **P < 0.01, and ns is not significant (Student’s *t* test). Data are representative of two experiments.

To investigate whether the functional defect in Breg cells from DOCK8 KO mice is intrinsic to these cells, we transferred CD45.2^+^ wild-type or DOCK8 KO bone marrow cells plus CD45.1^+^ bone marrow cells (4×10^6^ cells in a 1:1 mixture) into lethally-irradiated (two doses of 550 rads each) wild-type CD45.1^+^ recipients. Recipient mice were allowed 8 weeks to reconstitute before being challenged with OVA. We found that the percentage of CD45.2^+^IL-10^+^ B cells (**Figures 2A, B**) and CD45.2^+^CXCR5^+^ Tfh cells (**Supplemental Figure 1**) in the spleens of immunized chimeric mice harboring Dock8-deficient bone marrow cells decreased. However, B cells in the mixed chimeras retained some IL-10-producing functions (the frequency of IL-10^+^ B cells was about 0.5%, whereas that in OVA-immunized Dock8 KO mice was almost 0% (see **Figure 1G**), suggesting that IL-10 secretion by Dock8-deficient B cells may also be affected by other cells. These results indicate that the Breg defect in Dock8 deficiency is not entirely B cell-intrinsic.

### DOCK8^-/-^ CD4^+^ T Cells Impair IL-10 Production by B Cells

**Figure 3:**
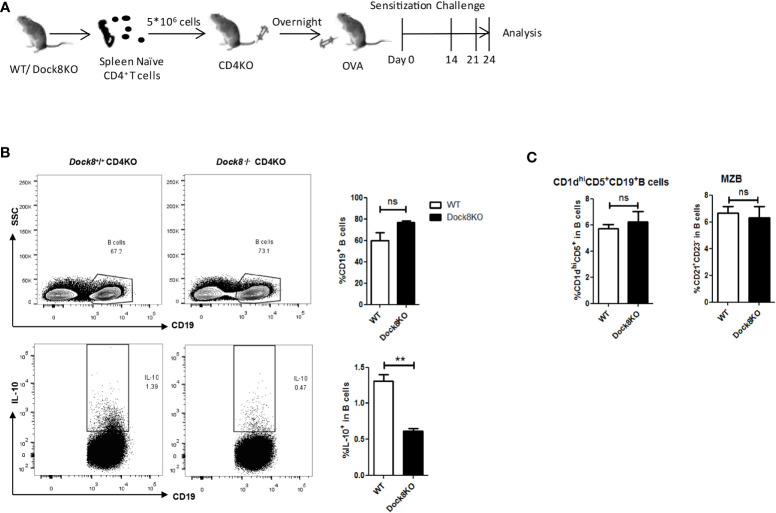
Dock8^-/-^ CD4^+^ T cell impairs IL-10 production by B cells. **(A)** Splenic CD4^+^ naïve T cells from wild-type (n = 4) or Dock8 KO mice (n = 4) were transferred into CD4 KO mice 1 day before immunization with OVA. Mice were sensitized with 5 μg OVA intraperitoneally on Day 0, followed by two oropharyngeal aspiration challenges (on Days 14 and 21). Mice were harvested 72 h after the second challenge. **(B)** The percentage of CD19^+^IL-10^+^ B cells in CD4 KO mice receiving Dock8^-/-^ CD4^+^ naïve T cells was lower than that in mice receiving wild-type CD4^+^ naïve T cells. **(C)** Dock8^-/-^ CD4^+^ T cells have no effect on the percentage of different Breg subtypes. **P < 0.01, and ns is not significant (Student’s *t* test). Data are representative of two independent experiments.

Breg cells require cognate interactions with IL-21-producing CD4^+^ T cells to secrete IL-10 *in vivo* (20), and abnormalities in CXCR5^+^CD4^+^ Tfh cells associated with DOCK8 deficiency has been described previously (29). Therefore, we performed adoptive transfer experiments to confirm the effect of Dock8^-/-^ CD4^+^ T cells on IL-10 production by B cells. CD4 KO mice received wild-type or Dock8 KO splenic CD4^+^ naïve T cells, followed by challenge with OVA. The adoptively transferred CD4^+^ T cells in the blood of CD4 KO mice were detected on day 7, day14 and day21 (**Supplemental Figure 2**). At 24 days post-immunization with OVA, we analyzed the IL-10^+^ Breg cell population in CD4 KO recipient mice (**Figure 3A**). The percentage of IL-10^+^ Breg cells in CD4 KO mice receiving Dock8 KO CD4^+^ naïve T cells was lower than that in mice receiving wild-type CD4^+^ naïve T cells (**Figure 3B**). There was no significant difference between two groups with respect to the percentage of CD1d^hi^CD5^+^ B cells or MZB cells (**Figure 3C**). Thus, Dock8 KO CD4^+^ naïve T cells do not affect the percentages of Breg subsets in recipient mice. These results suggest that loss of DOCK8 from CD4^+^ T cells attenuates IL-10 production by B cells *in vivo*.

### Supplementation With IL-21 Restores IL-10 Production by B Cells From DOCK8 KO Mice Both *In Vitro* and *In Vivo*

**Figure 4:**
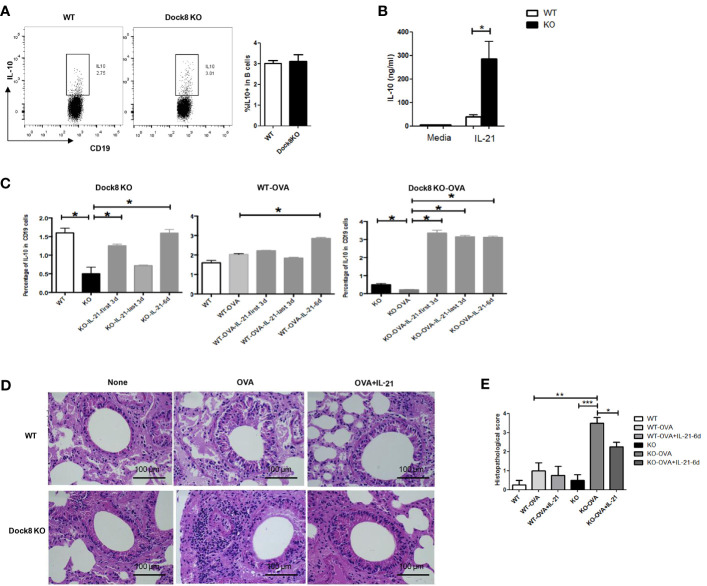
Exogenous IL-21 rescues IL-10 production by Breg cells in Dock8 KO mice both *in vitro* and *in vivo*. **(A)** Splenic B cells isolated from wild-type (n = 3) or Dock8 KO mice (n = 3) were co-cultured with IL-21 for 48 hours and the percentage of CD19^+^IL-10^+^ B cells was analyzed by FCM. **(B)** The IL-10 concentration in the supernatant was measured in an ELISA. **(C)** The percentage of CD19^+^IL-10^+^ B cells in wild-type (n = 3) and Dock8 KO mice (n = 3), which were sensitized with 5 μg OVA on Day 0, followed by two challenges (on Days 14 and 21). Finally, 20 ng of rmIL-21 was administered daily (for 3 or 6 days; Days 15–17, 18–20, or 15–20) into the nostrils. Mice were harvested 72 h after the second challenge. **(D)** Representative images of H&E stained lung tissue from each genotype in the OVA-induced allergic asthma model.”OVA+IL-21” images are from 6 days of IL-21 treatment. **(E)** Histopathological score for airway inflammation. *P < 0.05, **P < 0.01, ***P < 0.001, and ns is not significant (Student’s *t* test for **(A, B)**, ANOVA for **(C–E)**. Data in **(A, B, D)** are representative of three independent experiments. Data in **(C)** are representative of two experiments.

To confirm whether reduced IL-21 secretion by CD4^+^ T cells under conditions of Dock8 deficiency causes the functional defect in Breg cells, we co-cultured purified splenic B cells (2×10^6^) from wild-type or Dock8 KO mice with IL-21. After culture for 48 h, the IL-10 concentration in the supernatant was measured in an ELISA. Whereas the percentage of IL-10^+^ B cells in Dock8 KO mice was comparable with that in WT mice (**Figure 4A**), the concentration of IL-10 was higher (**Figure 4B**).

Next, to further examine the effect of IL-21 on IL-10 production by B cells *in vivo*, we exposed wild-type or Dock8 KO mice to intranasal rmIL-21 for 3 or 6 days. Surprisingly, the percentage of IL-10^+^ B cells in the spleen of Dock8 KO mice was almost comparable with that in wild-type mice (**Figure 4C**). Increased production of IL-10 by B cells was more obvious in Dock8 KO mice sensitized with OVA. At the same time, treatment with recombinant IL-21 provided Dock8 KO mice with marked protection from OVA-induced airway inflammation, accompanied by alleviation of inflammatory infiltration (**Figures 4D, E**). Whereas treatment with rmIL-21 fully restored the percentage of IL-10^+^CD19^+^ B cells in the spleens of Dock8 KO mice to WT levels (**Figure 4C**), it only partially alleviated airway inflammation (**Figure 4E**), suggesting that other cells might also play a role. Taken together, these data suggest that IL-21 plays a critical role in the normal function of Breg cells under conditions of Dock8 deficiency, and that exogenous IL-21 rescues defective IL-10 production by B cells in Dock8 KO mice.

### LPS-Driven, Not IL-21-Driven, STAT3 Phosphorylation Is Defective in Breg Cells From Dock8 KO Mice

**Figure 5:**
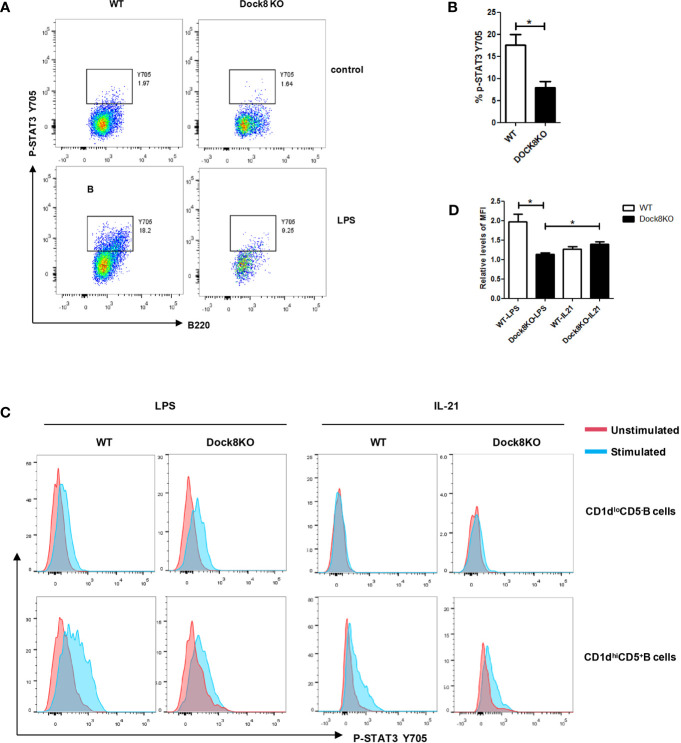
LPS-induced STAT3 phosphorylation, but not IL-21-induced STAT3 phosphorylation, is defective in Breg cells from DOCK8 KO mice. **(A, B)** STAT3 phosphorylation on Y705 in splenic B cells from wild-type (n = 3) and Dock8 KO mice (n = 3) after 3 hours of LPS stimulation. **(C, D)** STAT3 phosphorylation in CD5^+^CD1d^hi^ B cells and CD5^-^CD1d^lo^ B cells after stimulation with LPS for 3 hours or rmIL-21 for 30 minutes (n = 3 for both).The red line is the unstimulated samples, the blue line is the stimulated samples. Mean fluorescence intensity (MFI) levels of IL-10 in unstimulated samples of respective groups were defined as 1. *P < 0.05 (Student’s *t* test). Data in **(A)** are representative of three independent experiments, Data in **(C)** are representative of two independent experiments.

Because STAT3 phosphorylation is required for LPS-induced IL-10 production by B cells (18, 19), we examined this phenomenon in B cells from Dock8 KO mice stimulated with LPS. We found that levels of STAT3 phosphorylation in Dock8 KO mice were lower than those in wild-type mice (**Figures 5A, B**). Next, we examined STAT3 phosphorylation in CD1d^hi^CD5^+^ B cells (B10 cells) from Dock8 KO mice after stimulation with LPS or IL-21. We found no significant increase in STAT3 phosphorylation in Dock8^-/-^ CD1d^hi^CD5^+^ B cells after LPS stimulation (**Figure 5C**). By contrast, IL-21 caused comparable STAT3 phosphorylation in CD1d^hi^CD5^+^ B cells from Dock8 KO and wild-type mice (**Figures 5C, D**).

## Disscussion

Here, we present evidence that Dock8 deficiency impairs IL-10 production by Breg cells due to abnormalities in Dock8^-/-^ IL-21-producing CD4^+^ T cells. We also show that exogenous IL-21 rescues the function of Breg cells in Dock8 KO mice both *in vitro* and *in vivo*.

B cells play an important role in the pathogenesis of allergic diseases, in particular by secreting IgE. However, several phenotypic subsets have been identified as Breg cells; these cells exert immunosuppressive functions in allergic and inflammatory diseases *via* release of IL-10 (30). In murine models of allergic airway disease, Breg deficiency is associated with increased serum IgE levels, increased secretion of type 2 cytokines, and increased eosinophilia (31, 32). Patients with allergic asthma and rhinitis show a decrease in the percentage of IL-10-secreting CD19^+^CD24^hi^CD27^+^ Breg cells in response to LPS stimulation (14, 33). Injection of IL-10-producing CD9^+^ Breg cells into asthmatic mice normalizes airway inflammation and lung function by inhibiting Th2- and Th17-driven inflammation (34).

In the present study, we used an OVA-induced allergic asthma model based on Dock8 KO mice, as previously described (27). Model mice showed severe reductions in the percentage of IL-10-producing Breg cells, increased serum OVA-specific IgE levels, and increased inflammatory infiltration compared with wild-type mice and Dock8 KO mice not exposed to OVA. DOCK8 deficiency is a HIES; therefore, it is associated with a high incidence of allergic disease. Indeed, 71% of patients have allergic manifestations and 30% develop asthma (35). Among the three patients examined in the present study, one patient had food allergies and one had asthma. Due to the small number of enrolled patients, we were unable to test whether the number of IL-10-producing Breg cells differed between patients with and without allergies. Further studies should clarify whether Breg cells are involved in the onset of allergic disease under conditions of DOCK8 deficiency.

Our data also provide insight into the mechanism by which DOCK8 may cause a severe reduction in Breg cell numbers. IL-10 production by Breg cells requires LPS-induced TLR signaling (36). Nonetheless, IL-21- and CD40 dependent cognate interactions with T cells are also required for IL-10-producing Breg cells to optimally suppress inflammation and autoimmunity (20), and adding CD40L to the LPS cultures increased IL-10 producing B-cell frequencies in the health controls (**Supplemental Figure 3**). DOCK8-deficient patients may also have higher numbers of IL-10 producing B-cell if stimulated appropriately. All of these signals are instrumental to Breg expansion and function; thus dysfunction leads to impaired IL-10 production and susceptibility to inflammation and allergy. Because impaired expansion of CXCR5^+^CD4^+^ Tfh cells has been found in DOCK8 deficiency, we adoptively transferred DOCK8^-/-^ CD4^+^ T cells into CD4 KO mice and found that this impaired IL-10 production by B cells, which suggests that abnormalities of Dock8^-/-^CD4^+^ T cells affect the function of Breg cells. Subsequently, administration of rmIL-21 improved IL-10 production by Breg cells and ameliorated airway inflammation in Dock8 KO mice. All of these results confirm that IL-21-dependent interactions with T cells play a critical role in the normal function of Breg cells under conditions of DOCK8 deficiency. Reduced Breg cell numbers have also been observed in a variety of allergic diseases. Whether IL-21^+^ Tfh cells are also involved in the defect of Breg cells remains to be explored.

As reported previously, TLR-driven STAT3 phosphorylation was defective in B cells from DOCK8-deficient patients, but IL-21 driven STAT3 phosphorylation in B cells from DOCK8-deficient patients was comparable with that in HCs (6). Our data suggest that LPS-driven, but not IL-21-driven, STAT3 phosphorylation is defective in Breg cells from Dock8 KO mice. Thus, it is possible that IL-21 restores the Breg defect in DOCK8 deficiency by inducing normal STAT3 phosphorylation.

Recently identified functions of DOCK8 explain why its loss might result in allergy; for example, generation of IgE-promoting IL-13^+^ follicular helper T cells (3). These “Tfh13” cells produce IL-13 and IL-4, but downregulate production of IL-21. Consistent with this, our data suggest that defective IL-21^+^ CD4^+^ T cells might contribute to allergy in Dock8 KO mice, and that supplementation with IL-21 reduces airway inflammatory infiltration, as well as the number of serum IgE and IgE-producing B cells, in the OVA-induced allergic asthma model (27). Thus, IL-21 plays an important role in the pathogenesis of allergic asthma. However, mixed results have been reported regarding the impact of IL-21 in asthmatic mice; for example, studies suggest that IL-21 either promotes airway eosinophilia and type 2 immunity (21), or inhibits IgE production and decreases eosinophil recruitment into the airways (37). Tfh cells are the main cellular source of IL-21, which inhibits IgE class switch recombination in B cells by triggering STAT3 activation (38). Because IL-21 has a profound effect on IgE production, supplementation with IL-21 may rebalance the elevated IgE levels in patients with asthma. It is still not very clear why IL-21 signaling plays different roles in asthma; therefore, more studies are needed to provide definitive evidence.

In summary, we show here that DOCK8 regulates Breg function in both humans and mice. We propose that the absence of Bregs under conditions of DOCK8 deficiency contributes to allergy and chronic inflammation in these patients. Data from this study provide new insight into the potential design of Breg-based or IL-21-based therapeutic strategies for allergic diseases, including asthma in those with DOCK8 deficiency.

## Data Availability Statement

The original contributions presented in the study are included in the article/**Supplementary Material**. Further inquiries can be directed to the corresponding author.

## Ethics Statement

The studies involving human participants were reviewed and approved by Medical Ethics Committee of Children’s Hospital of Chongqing Medical University. Written informed consent to participate in this study was provided by the participants’ legal guardian/next of kin. The animal study was reviewed and approved by Medical Ethics Committee of Children’s Hospital of Chongqing Medical University.

## Author Contributions

JJ and XZ designed the study and wrote the manuscript. JJ, TQ, LiaZ, QL, JW, RD, LinZ, QZ, XL, and HW performed the experiments and analyzed the data. TQ and XZ followed-up the patients. All authors contributed to the article and approved the submitted version.

## Funding

This work was supported by the National Natural Science Foundation of China (81803140).

## Conflict of Interest

The authors declare that the research was conducted in the absence of any commercial or financial relationships that could be construed as a potential conflict of interest.

## Publisher’s Note

All claims expressed in this article are solely those of the authors and do not necessarily represent those of their affiliated organizations, or those of the publisher, the editors and the reviewers. Any product that may be evaluated in this article, or claim that may be made by its manufacturer, is not guaranteed or endorsed by the publisher.
