# Supplementary material for: IL-21 Rescues the Defect of IL-10-Producing Regulatory B Cells and Improves Allergic Asthma in DOCK8 Deficient Mice
Source: Front Immunol. 2021 Nov 15;12:695596. doi: 10.3389/fimmu.2021.695596 (PMC8636116; doi:10.3389/fimmu.2021.695596)
Supplement: Supplementary file 1 [file DataSheet_1.pdf]

Figure S1

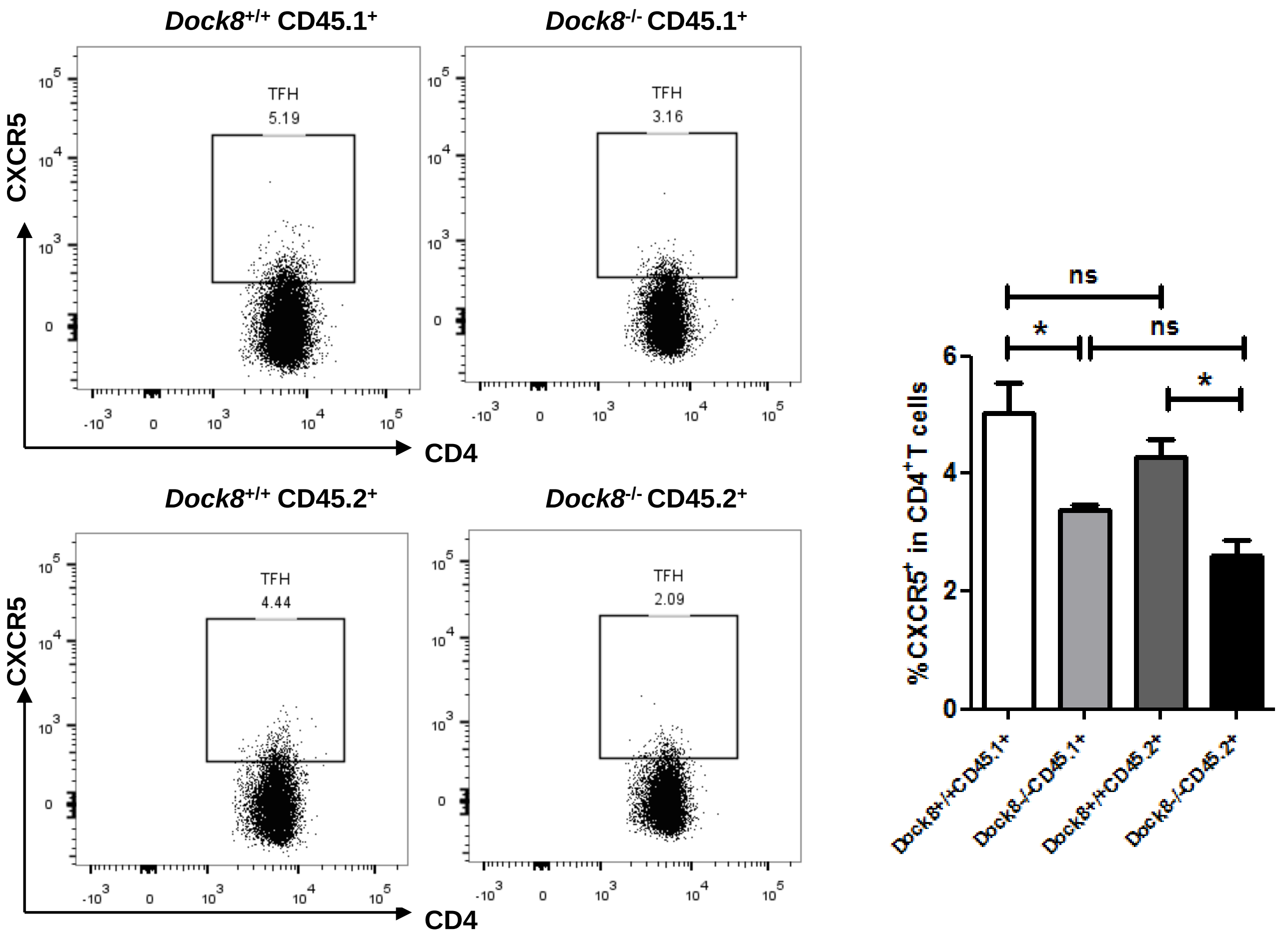

Figure S1. Flow cytometry analysis of CD45.2$^+$CXCR5$^+$ TFH cells in the splenocyte population from CD45.1$^+$ chimeric mice. CD45.2$^+$ wild-type or Dock8 KO bone marrow cells were transferred intravenously into lethally-irradiated CD45.1$^+$ wild-type recipients (n=3 for both) prior to immunization with OVA. *P <0.05 (Student's *t* test).

Figure S2

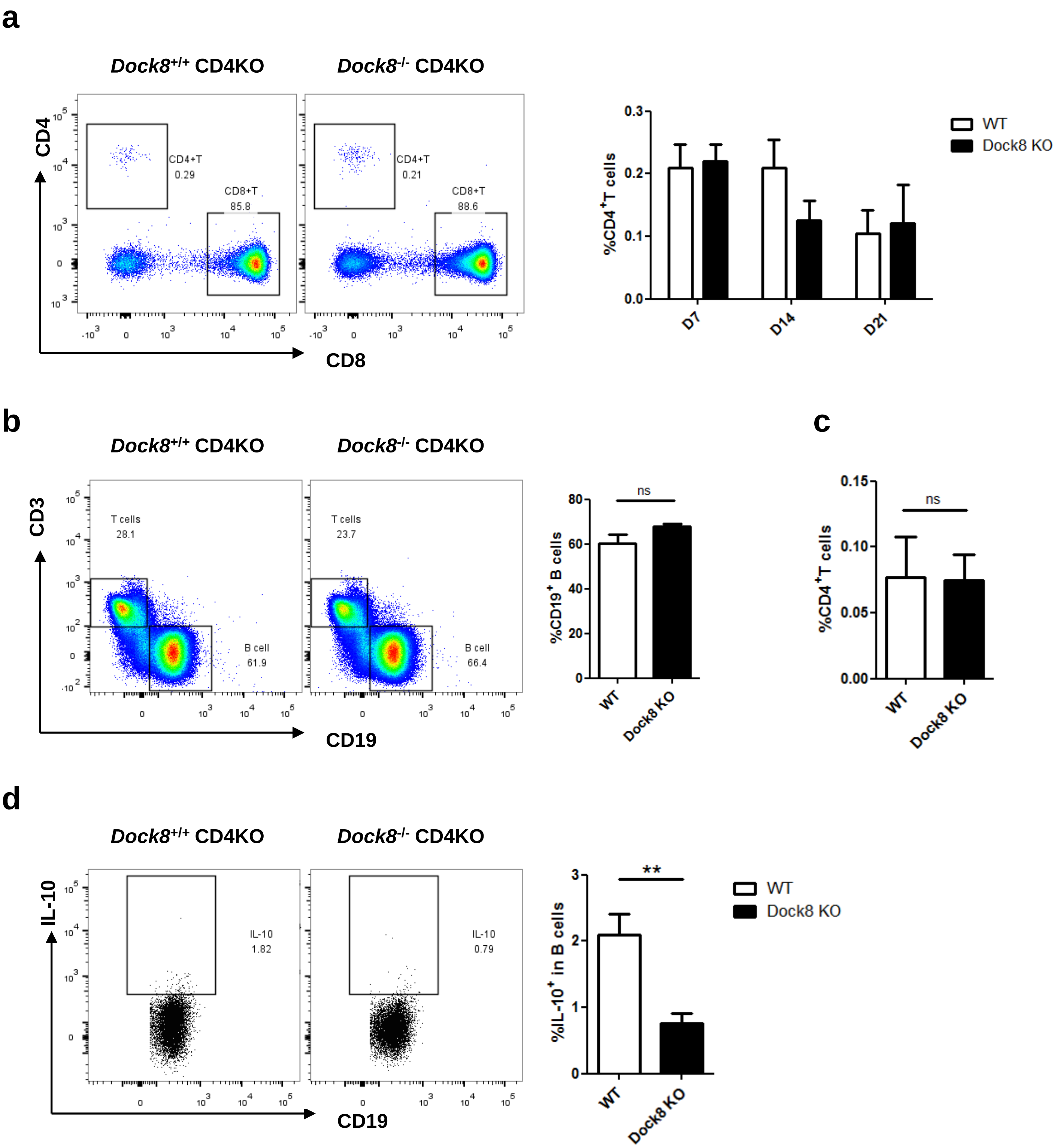

Figure S2. Flow cytometry of CD4$^+$ T cells in the blood of CD4KO mice (n=4) adoptively transferred with WT and *Dock8*$^{-/-}$ na ïve CD4$^+$ T cells. (A) Blood was taken from the tail vein of CD4KO mice on day 7, day14 and day 21 to detect the number of CD4$^+$ T cells. (B-D) Recipient mice were analyzed on Day 24 post-OVA immunization. **P <0.01 and ns is not significant.

Figure S3

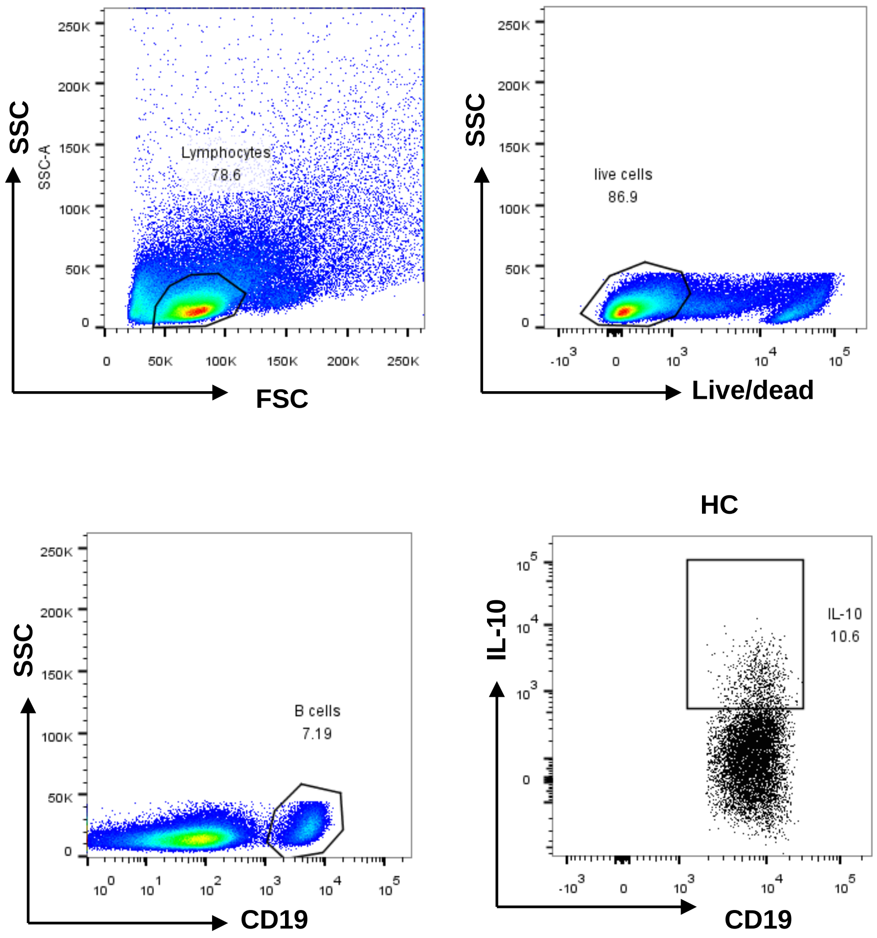

Figure S3. Flow cytometry analysis of IL-10 producing B cells from health control. The cells were treated with LPS and CD40L for 48 h; PMA, ionomycin and BFA were then added for the final 5 h of culture.
